# Cutaneous and Systemic Complications in Primary CD8+ Aggressive Epidermotropic Cytotoxic T-cell Lymphoma

**DOI:** 10.7759/cureus.100184

**Published:** 2025-12-27

**Authors:** Jacqueline Nikakis, Zahidul Islam, Bret-Ashleigh Coleman, Xiaotong Wang, Khurram Mehtabdin, Stanley Skopit

**Affiliations:** 1 Dermatology, Larkin Community Hospital, South Miami, USA; 2 Medicine, Peconic Bay Medical Center, Riverhead, USA; 3 Medicine, Baptist Health, Birmingham, USA; 4 Pathology, Northwell Health, New Hyde Park, USA; 5 Nephrology, Peconic Bay Medical Center, Riverhead, USA

**Keywords:** chemotherapy-induced adverse effects, cutaneous lymphoma complications, primary cutaneous cd8+ aggressive epidermotropic cytotoxic t-cell lymphoma, primary cutaneous t-cell lymphoma, renal-limited thrombotic microangiopathy

## Abstract

CD8+ aggressive epidermotropic cytotoxic T-cell lymphoma (CD8+ AECTCL) is an uncommon and highly aggressive variant of cutaneous T-cell lymphoma characterized by rapid systemic progression. We present the case of a 61-year-old man with CD8+ AECTCL who developed a methicillin-sensitive *Staphylococcus aureus *port-site infection while on duvelisib following prior gemcitabine/liposomal doxorubicin therapy. The patient had been diagnosed with CD8+ AECTCL 18 months prior and presented with acute erythema, swelling, and pain over his left anterior chest. Upon admission, the patient met sepsis criteria, and his port was subsequently removed in addition to the initiation of appropriate antibiotic therapy. During hospitalization, he developed nephrotic-range proteinuria and acute kidney injury requiring dialysis. Renal biopsy revealed diabetic nephropathy with findings consistent with renal-limited thrombotic microangiopathy (TMA), likely multifactorial from gemcitabine and duvelisib exposure compounded by sepsis and underlying lymphoma. Complement studies supported the activation of the alternative pathway, and after multidisciplinary discussion, eculizumab was initiated for TMA management. The patient completed inpatient eculizumab dosing and was discharged on outpatient infusions. His cutaneous lymphoma lesions improved with a short course of oral prednisone as directed by oncology. At discharge, he was clinically stable on dialysis with plans for ongoing outpatient care; subsequent follow-up showed stable renal function. This case highlights the intersection between aggressive cutaneous lymphoma, infection, and chemotherapy-induced vascular injury, emphasizing the importance of early recognition of systemic complications in CD8+ AECTCL and the need for a multidisciplinary approach to optimize patient outcomes.

## Introduction

Primary cutaneous T-cell lymphomas (CTCLs) are rare non-Hodgkin lymphomas characterized by the proliferation of malignant T cells in the skin. Among these, primary cutaneous CD8+ aggressive epidermotropic cytotoxic T-cell lymphoma (CD8+ AECTCL) is an uncommon and highly aggressive variant, first described by Berti et al. in 1999, and accounting for roughly 1% of all CTCLs [1]. Patients typically present with widespread plaques, nodules, or ulcerated masses, reflecting the disease's destructive nature. Histopathologic hallmarks include epidermotropic infiltrate with irregular hyperchromatic nuclei and epidermal necrosis [2]. The disease course is rapidly progressive, with median survival ranging from 12 to 32 months [3], emphasizing its poor prognosis and the urgency of timely intervention.

The etiology of CD8+ AECTCL remains incompletely understood. Emerging evidence implicates the dysregulation of signaling pathways involved in T-cell proliferation and survival, including JAK2 and SH2B3, in disease pathogenesis [4]. However, no unifying pathogenic mechanism has been identified, and the initiating events leading to malignant transformation remain unclear. Likewise, no consistent predisposing risk factors have been established, although epidemiologic data suggest that CD8+ AECTCL predominantly affects older adults, most commonly presenting in the seventh decade of life.

Histopathologic evaluation is central to diagnosis and demonstrates the striking epidermotropism of atypical cytotoxic T-lymphocytes. Characteristic features include band-like or diffuse dermal infiltrates composed of enlarged lymphocytes with irregular nuclei, marked pagetoid spread into a thickened, acanthotic epidermis, and areas of focal necrosis [5]. Immunophenotypically, tumor cells are typically CD3+, CD8+, and CD45RA+, with frequent loss of pan-T-cell markers such as CD2 and CD5, along with a high Ki-67 proliferation index reflecting the tumor's aggressive behavior [3]. Collectively, these clinicopathologic findings underscore the diagnostic complexity of CD8+ AECTCL and highlight the importance of maintaining a high index of suspicion in patients presenting with rapidly progressive or ulcerative cutaneous lesions.

Clinically, CTCLs can resemble common papulosquamous disorders, which can delay accurate diagnosis and complicate management [6]. Patients are particularly susceptible to infections due to damage to the skin barrier caused by the lymphoma and its treatments, a risk that is further increased in immunosuppressed individuals. Our case illustrates the severe skin and systemic complications that can occur in CD8+ AECTCL, including infection at a chemotherapy port site. This situation highlights the complex relationship between the lymphoma, secondary infections, and systemic treatment-related complications such as thrombotic microangiopathy (TMA), demonstrating the need for careful monitoring and a multidisciplinary approach.

Therapeutic options for CD8+ AECTCL remain limited, reflecting the aggressive nature of this lymphoma. Conventional approaches, including multi-agent systemic chemotherapy, radiation therapy for localized lesions, and allogeneic hematopoietic stem cell transplantation in select patients, are employed with variable success, but durable remissions are uncommon, and overall outcomes remain poor [7,8]. These limitations highlight the need for careful monitoring and individualized treatment planning, as well as the continued exploration of novel therapies to improve patient outcomes. With the emergence of targeted agents, including duvelisib for refractory disease, recognition of both dermatologic and systemic effects of aggressive CTCL is critical to optimize management [9]. This case emphasizes the importance of promptly identifying potentially life-threatening presentations in patients with indurated or ulcerated skin lesions, particularly when systemic symptoms suggest a broader underlying disease process or treatment-related complications. By exploring the interplay of cutaneous disease, infection, and chemotherapy, this report adds to the literature, guiding the management of aggressive CTCL.

## Case presentation

A 61-year-old Caucasian man with a history of primary cutaneous CD8+ AECTCL, type 2 diabetes mellitus, hypertension, and coronary artery disease presented with acute erythema, swelling, and pain over his left anterior chest. He had been diagnosed with CD8+ AECTCL 18 months earlier, and a left anterior chest wall port had been placed for chemotherapy access. Initial treatment with gemcitabine and liposomal doxorubicin was discontinued several months prior to admission and followed by ruxolitinib. With subsequent disease progression, gemcitabine and doxorubicin were reinitiated; the last gemcitabine dose was administered approximately six weeks before presentation and the last doxorubicin dose three weeks prior. Single-agent duvelisib was initiated less than one week before hospital admission.

**Figure 1:**
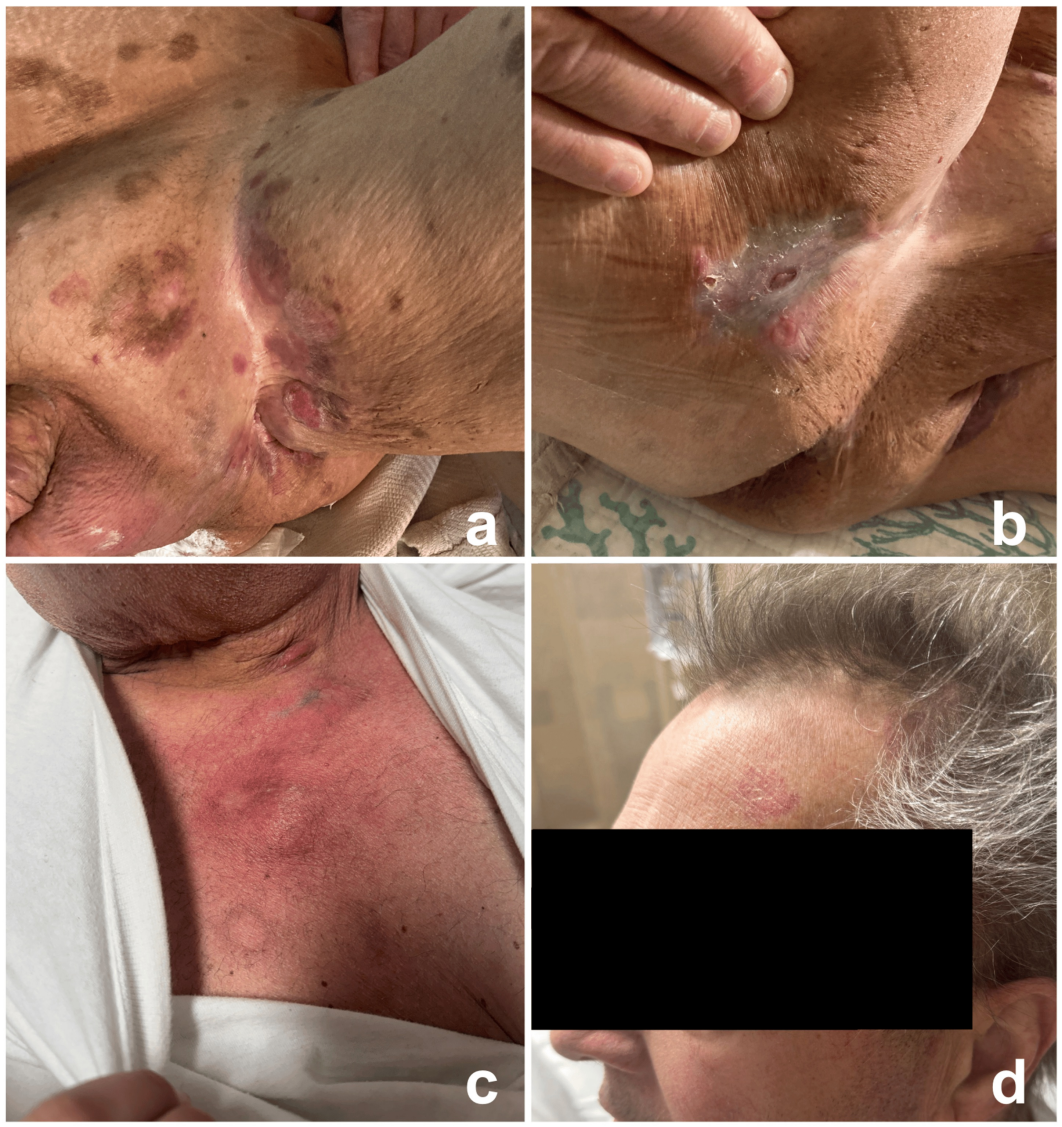
Cutaneous manifestations (a) Multiple scattered erythematous and dark brown (hyperpigmented) plaques and ulcerations in the left groin. (b) A 4×2 cm atrophic plaque with central ulceration on the right buttock. (c) A 6×6 cm indurated, warm, erythematous plaque with tenderness around the left chest port site. (d) A 2×1 cm scaly erythematous plaque on the left superior forehead.

Four days prior to admission, he developed progressive erythema, warmth, and tenderness surrounding the port site. Initially afebrile, he subsequently experienced malaise and subjective fever. On examination, there was a 6×6 cm indurated, warm, erythematous plaque with tenderness around the port site consistent with an infectious process (Figure 1).

On admission, he was febrile to 100.3°F and tachycardic. Laboratory evaluation revealed leukocytosis (WBC 11.3×10^9^/L; reference range: 4.5-11.0×10^9^/L), moderate anemia, and acute kidney injury (creatinine 2.1 mg/dL; reference range: 0.6-1.3 mg/dL). Blood cultures grew methicillin-sensitive *Staphylococcus aureus *(MSSA) from both port and peripheral samples. He was started on broad-spectrum IV antibiotics, later narrowed to cefazolin, which he completed for four weeks. The port was removed, yielding purulent drainage consistent with abscess formation; wound cultures also grew MSSA.

Despite adequate source control and antimicrobial therapy, he developed progressive edema, hypoalbuminemia, and worsening renal dysfunction. Urinalysis revealed nephrotic-range proteinuria (>18 g/24 h; normal <150 mg/24 h) with hematuria. Infectious workup for secondary causes was negative (HIV, hepatitis B/C, syphilis, and other viral serologies). Autoimmune and paraneoplastic panels were unrevealing.

During hospitalization, the patient's course was complicated by an upper respiratory tract infection. Polymerase chain reaction (PCR) testing was positive for influenza A, H1N1 flu, and human metapneumovirus. These findings were considered incidental, as the patient exhibited only mild respiratory symptoms, required no antiviral therapy, and showed no evidence of virus-associated renal injury or exacerbation of systemic inflammation. Supportive care was provided, and the co-infections did not temporally or mechanistically correlate with the progression of his renal dysfunction.

**Figure 2:**
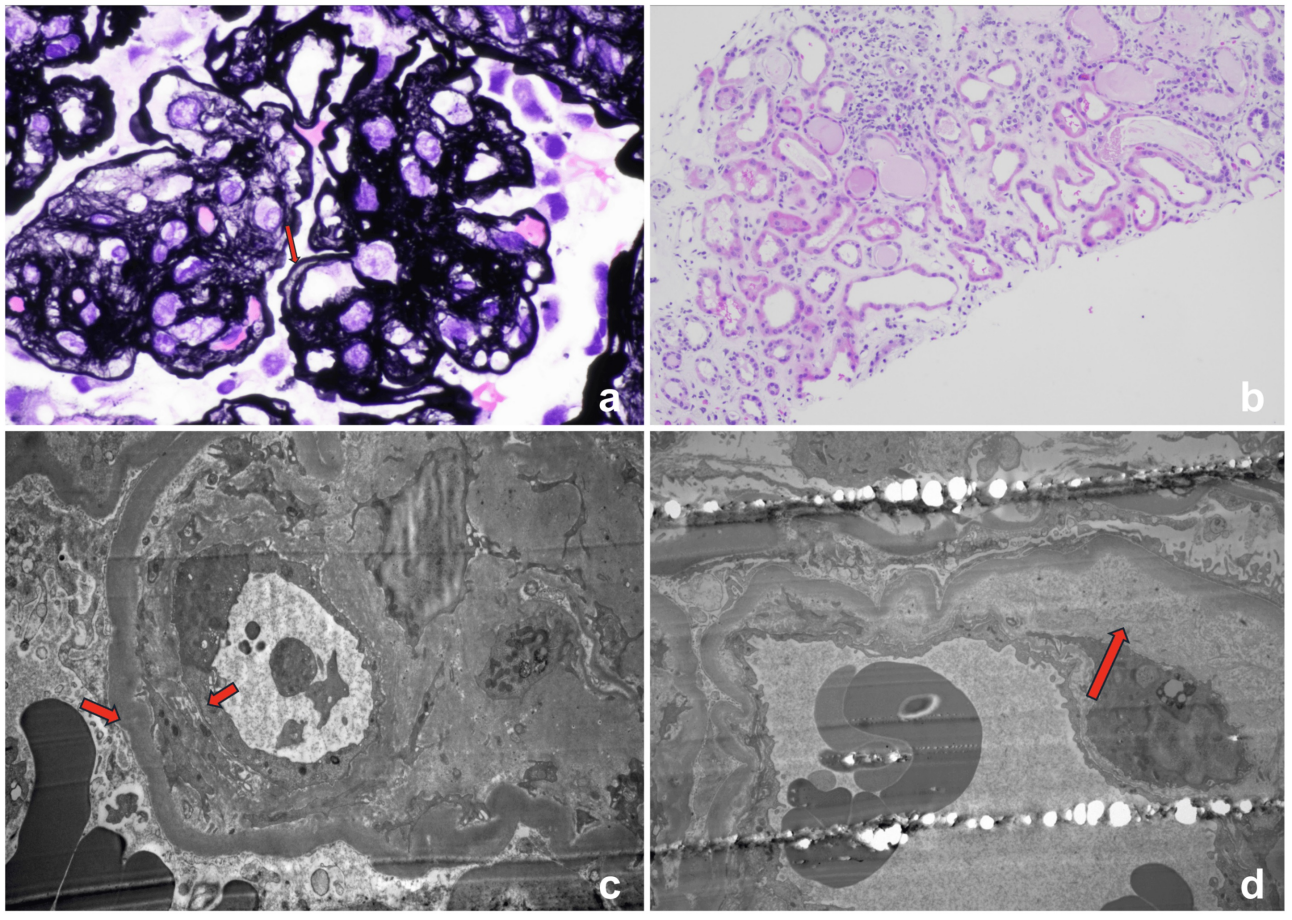
Renal histology Pathologic features of TMA identified include (a) duplication of glomerular capillary walls (arrows) and (b) acute tubular injury. EM exam reveals (c) segmental duplication of GBM and (d) subendothelial expansion by electron-lucent material. TMA: thrombotic microangiopathy; EM: electron microscopy; GBM: glomerular basement membranes

Renal function continued to decline throughout admission, with creatinine peaking at 7.3 mg/dL and oliguria requiring the initiation of hemodialysis. A CT-guided right renal biopsy demonstrated diabetic nephropathy with nodular mesangial sclerosis (Renal Pathology Society (RPS) class III), in addition to the duplication of glomerular capillary walls (Figure 2A) and acute tubular injury (Figure 2B). Immunofluorescence studies were negative for immune-complex mediated glomerulonephritis. Electron microscopy (EM) revealed the segmental duplication of glomerular basement membranes (GBM) without immune deposits (Figure 2C) and subendothelial expansion by electron-lucent material (Figure 2D). These structural changes, particularly capillary wall duplication and subendothelial lucency, indicate ongoing endothelial injury consistent with TMA rather than diabetic nephropathy alone. The lack of immune deposits helps rule out immune-complex glomerulonephritis, and the presence of acute tubular injury supports ischemia from microvascular compromise. Taken together, the biopsy was most consistent with a renal-limited TMA, presumed multifactorial and likely related to gemcitabine.

The patient's cutaneous lesions, including the port-site infection and areas of prior lymphoma involvement, gradually improved with ongoing wound care. He was subsequently transferred to another center for further oncologic management of CD8⁺ AECTCL. Complement studies obtained more than one week after admission demonstrated a C3 level of 96 mg/dL and C4 level of 17 mg/dL, followed one week later by a decline in C3 to 80 mg/dL with a stable C4 of 18 mg/dL (reference ranges: C3, 90-180 mg/dL; C4, 10-45 mg/dL). Although complement levels were low-normal, these findings did not exclude complement-mediated TMA. After multidisciplinary discussion, eculizumab was initiated approximately three to four weeks after admission for the management of suspected complement-mediated TMA. The patient completed inpatient eculizumab induction and was discharged on a scheduled outpatient infusion regimen. His cutaneous lymphoma lesions also improved following a short course of oral prednisone per oncology recommendations. At discharge, he remained dialysis-dependent but clinically stable, with coordinated outpatient follow-up arranged with nephrology, oncology, wound care, and the continuation of complement inhibitor therapy. During a subsequent hospitalization, renal function showed improvement, with serum creatinine stabilizing at approximately 2 mg/dL.

## Discussion

CD8+ AETCL is a rare and highly aggressive variant of CTCL, characterized by rapid progression and challenging management. Our case illustrates the multifaceted cutaneous and systemic complications in CD8+ AETCL. The initial port-site infection with MSSA highlights the vulnerability of the skin barrier in these patients. Lymphomatous infiltration and chronic ulceration compromise epidermal integrity, creating a local environment susceptible to microbial invasion. This vulnerability is compounded by systemic chemotherapy, which disrupts the epidermis and alters the skin microbiota, triggering inflammation and immune dysregulation [10,11]. Patients frequently develop xerosis and pruritus within weeks of initiating treatment, which can progress to painful fissures or asteatotic eczema, providing additional portals for infection. Reductions in skin hydration, sebum production, and overall barrier function further exacerbate cutaneous susceptibility, particularly in older adults or those with pre-existing skin conditions [12]. These observations emphasize the importance of proactive barrier management and vigilant monitoring to prevent infections in immunocompromised patients. 

Renal adverse events represent a significant source of morbidity in this population, with TMA representing a severe manifestation. TMA is characterized by endothelial injury, platelet aggregation, and microvascular thrombosis, which can result in acute kidney injury and nephrotic-range proteinuria [13]. Clinically, TMA is often classified by thrombocytopenia, schistocytes on peripheral smear, and microangiopathic hemolytic anemia with elevated lactate dehydrogenase, elevated indirect bilirubin, reduced haptoglobin, and increased reticulocyte count [14]. Recognizing these characteristic laboratory abnormalities is critical, as they guide clinicians in identifying TMA early and understanding the renal injury that follows. In our case, the development of nephrotic syndrome with biopsy-proven TMA was consistent with chemotherapy-associated vascular injury. Gemcitabine is a well-recognized cause of secondary TMA, directly inducing endothelial injury and microvascular thrombosis [15]. In addition to direct chemotherapeutic toxicity, immune-mediated endothelial damage may also contribute to TMA. Duvelisib, through dual PI3K-δ/γ inhibition, may promote TMA by decreasing regulatory T cells, increasing Th17 activation, and inducing systemic inflammation that drives endothelial injury and microthrombi formation [16,17]. Clarifying the mechanism of TMA is challenging but necessary, as treatment decisions depend on the specific underlying cause; once the etiology is identified, targeted interventions can be implemented to mitigate renal injury. In our patient, evidence of complement pathway activation guided the use of eculizumab, which resulted in a notable improvement in serum creatinine from 7.3 to 5.6 mg/dL over 19 days, highlighting the growing role of complement inhibition in secondary TMA [18,19]. Despite this partial renal recovery, the patient remained dialysis-dependent, emphasizing the long-term morbidity of renal complications.

In addition to medication-induced endothelial injury, the patient's various comorbidities likely further increased susceptibility to renal-limited TMA. Sustained elevations in blood pressure create significant shear stress in renal arterioles, leading to ischemia of the juxtaglomerular apparatus and activation of the renin-angiotensin system. Angiotensin II exerts direct vasculotoxic effects, generating a self-perpetuating cycle of endothelial injury and microvascular thrombosis that ultimately creates TMA lesions [20]. In addition, long-standing hyperglycemia compounds this vulnerability through multiple pathways, including the generation of advanced glycation end products, reactive oxygen species, and activation of the protein kinase C (PKC) and hexosamine pathways. These mechanisms reduce nitric oxide bioavailability and impair endothelial function, diminishing the vasculature's natural antithrombotic properties and increasing susceptibility to thrombotic injury [21,22]. These underlying factors, combined with gemcitabine-induced endothelial toxicity and duvelisib-associated immune dysregulation, likely created a highly pro-thrombotic microvascular environment leading to the development of TMA. 

Our case also exemplifies the necessity of a multidisciplinary approach in managing patients with CD8+ AETCL [23]. Coordinated care across dermatology, oncology, infectious disease, nephrology, and other specialties enables the early recognition of complications, enhanced therapeutic decision-making, and timely intervention when systemic toxicities arise. Such collaboration not only reduces the risk of delayed diagnoses or fragmented care but also enables the proactive management of cutaneous and systemic manifestations of disease [24,25]. Ultimately, multidisciplinary collaboration ensures that the diverse aspects of patient care are integrated, reducing the risk of oversight and ultimately supporting improved clinical outcomes.

## Conclusions

CD8+ AETCL is a rare and aggressive cutaneous lymphoma with multifaceted complications that extend beyond the skin to involve systemic organs. This case illustrates how cutaneous infections and chemotherapy-related renal toxicities can converge to produce life-threatening complications, emphasizing the necessity of early recognition and intervention. Optimal management requires a coordinated, multidisciplinary strategy, integrating dermatologic, oncologic, and nephrologic expertise to address both primary disease and secondary adverse events. Careful clinical monitoring for systemic manifestations in patients presenting with indurated or ulcerative lesions, particularly in the context of immunosuppressive therapy, is essential to improve outcomes and mitigate morbidity in this high-risk population.
